# Stress Reshapes the Physiological Response of Halophile Fungi to Salinity

**DOI:** 10.3390/cells9030525

**Published:** 2020-02-25

**Authors:** Yordanis Pérez-Llano, Eya Caridad Rodríguez-Pupo, Irina S. Druzhinina, Komal Chenthamara, Feng Cai, Nina Gunde-Cimerman, Polona Zalar, Cene Gostinčar, Rok Kostanjšek, Jorge Luis Folch-Mallol, Ramón Alberto Batista-García, María del Rayo Sánchez-Carbente

**Affiliations:** 1 Center of Research on Cell Dynamics, Autonomous University of the State of Morelos, Morelos 62210, Mexico; yordanis.perezllano@yahoo.com (Y.P.-L.); eyarguez2013@gmail.com (E.C.R.-P.); 2 Institute of Chemical, Environmental and Bioscience Engineering (ICEBE), TU Wien, 1060 Vienna, Austria; irina.druzhinina@njau.edu.cn (I.S.D.); komal.chentamara@gmail.com (K.C.); fengcai@njau.edu.cn (F.C.); 3 Fungal Genomics Group, Nanjing Agricultural University, Nanjing 210095, China; 4 Department of Biology, Biotechnical Faculty, University of Ljubljana, SI-1000 Ljubljana, Slovenia; Nina.Gunde-Cimerman@bf.uni-lj.si (N.G.-C.); polona.zalar@bf.uni-lj.si (P.Z.); Cene.Gostincar@bf.uni-lj.si (C.G.); rok.kostanjsek@bf.uni-lj.si (R.K.); 5 Laboratory of Molecular Biology of Fungi, Center for Research on Biotechnology, Autonomous University of the State of Morelos, Morelos 62210, Mexico; jordi@uaem.mx

**Keywords:** *Aspergillus sydowii* genome, fungal cell wall, compatible solutes, halophilic fungi, hydrophobins, fungal transcriptomics, osmotic stress

## Abstract

(1) Background: Mechanisms of cellular and molecular adaptation of fungi to salinity have been commonly drawn from halotolerant strains and few studies in basidiomycete fungi. These studies have been conducted in settings where cells are subjected to stress, either hypo- or hyperosmotic, which can be a confounding factor in describing physiological mechanisms related to salinity. (2) Methods: We have studied transcriptomic changes in *Aspergillus sydowii*, a halophilic species, when growing in three different salinity conditions (No NaCl, 0.5 M, and 2.0 M NaCl). (3) Results: In this fungus, major physiological modifications occur under high salinity (2.0 M NaCl) and not when cultured under optimal conditions (0.5 M NaCl), suggesting that most of the mechanisms described for halophilic growth are a consequence of saline stress response and not an adaptation to saline conditions. Cell wall modifications occur exclusively at extreme salinity, with an increase in cell wall thickness and lamellar structure, which seem to involve a decrease in chitin content and an augmented content of alfa and beta-glucans. Additionally, three hydrophobin genes were differentially expressed under hypo- or hyperosmotic stress but not when the fungus grows optimally. Regarding compatible solutes, glycerol is the main compound accumulated in salt stress conditions, whereas trehalose is accumulated in the absence of salt. (4) Conclusions: Physiological responses to salinity vary greatly between optimal and high salt concentrations and are not a simple graded effect as the salt concentration increases. Our results highlight the influence of stress in reshaping the response of extremophiles to environmental challenges.

## 1. Introduction

Ubiquitous filamentous fungus *Aspergillus sydowii* is mainly known as a pathogen of corals, which causes tissue lesions and darkening by melanization [1,2,3]. Additionally, this species can be found in hypersaline habitats, such as waters of the salterns [4,5] and dried foods [6]. However, it has been also isolated from various terrestrial niches, such as soil from near-Arctic to tropical regions [2,7,8] and decaying plant matter [9]. Interestingly, terrestrial isolates were consistently not pathogenic for corals, indicating a distinct ecotype, although genetic and metabolic signatures used so far did not confirm this distinction [2,10].

*A. sydowii* strains have been studied for their biotechnological potential as producers of secondary metabolites [11] and plant biomass-degrading enzymes [8,12,13]. A recent transcriptomic analysis of an *A. sydowii* isolate from Antarctic microalgae showed its ability to produce lignin-degrading enzymes and to grow on lignin as the sole carbon source [7].

We isolated a halophilic strain of *A. sydowii* in the search for a fungal model with two distinctive characteristics: the ability to grow in high salinity (up to 2.0 M NaCl) and the capacity to degrade plant biomass under these conditions. Due to its extremotolerant ecology and biotechnological potential, *A. sydowii* emerged as a prospective fungal model for the analysis of molecular adaptations to saline conditions. In fungi, these adaptations differ in many ways from adaptations of bacteria and archaea and have been described more extensively in Basidiomycota yeast and filamentous fungi [14], although some studies have also been conducted in filamentous Ascomycota, such as other Aspergilli [15,16,17,18]. The study aimed to identify, using transcriptomics, the mechanisms that mediate the halotolerance of *A. sydowii* at both optimal and extreme salinity. To date, this important distinction has not received sufficient attention, either in fungi or in prokaryotes [19]. Using the genome-wide transcriptomic approach, we have been able to delineate the osmoprotective strategies of a halophile in optimal growth conditions from responses triggered by osmotic stress.

## 2. Materials and Methods

### 2.1. Strain and Culture Conditions

The strain *Aspergillus sydowii* BMH-0004 was isolated at the Laboratory of Molecular Biology of Fungi at the Center for Research on Biotechnology (CEIB), Autonomous University of the State of Morelos (UAEM), Cuernavaca, Mor, Mexico. It was isolated from sugarcane bagasse and initially described by Batista-García et al., (2014) [20] as *A. caesiellus* H1. This strain was deposited in the Fungal Culture Collection of the CEIB (UAEM, Cuernavaca, Mexico) with reference number BMH-0004; in the Technological University Collection of Industrially relevant Microorganisms (TUCIM) (Vienna, Austria) with reference number 6524; and the Ex Culture Collection of the Infrastructural Centre Mycosmo (MRIC UL) (Ljubljana, Slovenia) with number EXF-12860.

To describe the morphology of the strain, the fungus was grown at 25 °C on malt extract agar (MEA), Czapek yeast extract agar (CYA), and creatine-sucrose agar (CREA) for 7 days. Additionally, the strain was grown at 30 and 37 °C on CYA and 25 °C on saline CYA (CYAS) with 5% NaCl. Colony diameter, color, texture, production of soluble pigments, and exudates were evaluated in all culture conditions. Conidia and conidiophores were visualized for colonies grown at 25 °C on MEA.

For RNA extraction, the strain was cultivated on a semi-solid fermentation of autoclaved (121 °C for 15 min) wheat straw. Three plugs of fresh mycelium grown on PDA without NaCl were inoculated onto 3.0 g of wheat straw soaked with thirty milliliters of Voegel’s medium (without NaCl (No NaCl) and with 0.5 M NaCl or 2.0 M NaCl) in 25.0-cm petri dishes and cultured for 7 days at 28 °C without agitation. For the relative quantification of selected genes by qPCR, an additional group cultured with 1.0 M NaCl in the medium was also considered. The mycelium was collected by adding 15 mL of diethyl-pyrocarbonate (DEPC)-treated water to recover the biomass from the petri dishes and vortexed in sterile conical tubes. This procedure was repeated three times, and the resulting washing liquid was centrifuged to obtain a pellet of cells. Triplicate cultures were obtained for RNA extraction and transcriptome profiling.

### 2.2. DNA Extraction, PCR Amplification, and Sequencing

Genomic DNA was extracted from PDA-grown mycelium using standard phenol:chloroform procedure. Primers for internal transcribed spacer (ITS) rDNA, RNA polymerase II second-largest subunit gene (*rpb2*), β-tubulin gene (*benA*), and calmodulin gene (*cam*) were used as recommended for classification of *Aspergillus* species [21], and PCR products were purified from 1% agarose gel using the GeneJET Gel Extraction kit (ThermoFisher Scientific, Waltham, MA, USA). Sanger sequencing of the amplicons was performed with the forward primers used for amplification, and the sequencing service was provided by Microsynth (Vienna, Austria).

### 2.3. Molecular Phylogeny

First, the sequences of ITS, *rpb2*, *benA*, and *cam* were submitted to a sequence similarity search by BLASTn against the NCBI nucleotide database. Most similar sequences and the corresponding published voucher DNA barcodes for the reference species [21] were retrieved. Sequences of *A. multicolor* NRRL 4775, *A. nidulans* NRRL 187, and *A. caesiellus* NRRL 5061 were chosen as outgroups (Supplementary Table S1). Multiple sequence alignments were created using the MUSCLE v3.8.425 [22] tool integrated into AliView [23]. Consecutively, a concatenated set of alignment was assembled using SEAVIEW [24] and realigned using MUSCLE in AliView. Where necessary, GBlocks [25] with less stringent conditions allowing smaller final blocks, gap position within the final blocks, and less strict flanking positions were used to remove ambiguous fragments (Supplementary Table S2). Maximum likelihood trees based on their specific best substitution model, as obtained using SMS, were created using PhyML tool (http://www.atgc-montpellier.fr/phyml-sms/) (Supplementary Figure S1). One thousand replicates were run to calculate bootstrap supports. Alternatively, the concatenated alignment with 88,625 nucleotides was subjected to Bayesian analysis using program MrBayes v3.2.5 [26] with an unconstrained GTR + I + G nucleotide substitution model applied to all loci. The chain was run for 3 million generations by applying two simultaneous, completely independent analyses starting from different random trees using three heated chains and one “cold” chain. Once the analyses were finished, 22,500 trees were summarized after discarding the first 25% of 30,000 trees generated, and a consensus tree was obtained. The trees were visualized and mid-point rooted using FigTree v1.4.22 (http://tree.bio.ed.ac.uk/software/figtree/). Further image improvement was performed in CorelDraw Graphics Suite X8 (Corel corporation, ON, Canada).

### 2.4. RNA Extraction, Library Construction, and Sequencing

Samples were ground with liquid nitrogen, and RNA purification was performed by the Tri-Reagent method (Sigma-Aldrich, San Louis, MO, USA). Libraries were constructed with the TruSeq Stranded mRNA HT kit (Illumina, San Diego, CA, USA), and sequencing was performed by the Mass Sequencing Unit of the Biotechnology Institute (UNAM, Cuernavaca, Mor, México) with a NextSeq 500 Sequencer (Illumina, San Diego, CA, USA) using 150 cycles of pair-end readings.

### 2.5. Transcriptome Assembly and Transcript Quantification

Sequence reads were filtered/trimmed by quality using FASTQC/Trimmomatic (cutoff: Q20), and *de novo* assembly was performed with Trinity [27]. The assembled transcriptome was mapped back to the *A. sydowii* genome for quality control purposes, which rendered a 90.4% overall similarity and approximately 73% coverage. Functional annotation of transcript sequences was achieved by BLAST against *nr* NCBI RefSeq and UniProt databases using Trinotate software [28]. Pfam, GO, and KOG term annotation, the prediction of signal peptide and transmembrane region (SignalP v4.1 [29]), were also performed. When considering membrane transporters, deducing the function of the protein from its sequence is often difficult. This is especially true for metal cation transporters, where substrate specificity and selectivity cannot be unambiguously predicted. To partially alleviate this problem, the Transporter Classification Database (TCDB: http://www.tcdb.org) was used to refine the annotations of transcripts belonging to transporter encoding genes of *A. sydowii*, and substrate specificity of DE transcripts was predicted using the TrSSP tool (http://bioinfo.noble.org/TrSSP/).

The mapping of reads to the assembled transcriptome was done by SMALT (http://www.sanger.ac.uk/science/tools/smalt-0). With this alignment, expression levels were quantified using Samtools [30]. Expression levels were reported in transcripts per million (TPM) [31,32], obtained by the RSEM program [33] using mappings obtained by Bowtie2 software [34]. Cumulative transcript expression values were obtained by adding the TPM values of transcripts mapped to functionally identified orthologous genes, and differences in cumulative expression between groups were assessed by two-way ANOVA with posterior Tukey’s multiple comparison test (α = 0.05).

### 2.6. Differential Transcript Expression Analysis

In order to perform the differential expression analysis, transcripts with a low number of read counts (CPM < 2) were filtered, aiming to increase the statistical strength of the prediction of differentially expressed transcripts [31]. As shown in Supplementary Figure S2a, the deletion of these transcripts allows the clustering (nonsupervised hierarchical clustering method) of sample profiles according to the treatment groups, indicating that it reduces the noise (i.e., variability that is not associated with biological conditions). Removal of these transcripts does not affect the genes identified, as differentially expressed (DE) (Supplementary Figure S2b). Based on this nonsupervised hierarchical clustering analysis, we removed a sample replicate belonging to the group 0.5 M NaCl from subsequent steps, as it is an outlier.

Expression data were normalized using the RUVseq algorithm [35] for the removal of variation between samples, and differential transcript expression analysis was performed using DESeq2 [36]. Transcripts with logFC > 2.0 and FDR < 0.05 were considered as differentially expressed (DE).

Gene ontology enrichment was determined by the over-representation analysis (ORA) method with a Fisher’s statistical test using the topGO package (https://bioconductor.org/). Pathway enrichment analysis was done using the BlastKOALA tool from KEGG database.

### 2.7. qPCR and Transcriptome Validation

For validation of RNA sequencing results, 1 µg of total RNA isolated as previously described from semi-solid wheat straw fermentation was treated with DNase I (Thermo Fisher Scientific, Waltham, MA, USA) and used for cDNA synthesis with the RevertAid First-Strand Synthesis kit (Thermo Fisher Scientific, Waltham, MA, USA) using oligo (dT) nucleotides. Two-step quantitative PCR (qPCR) reactions were carried out in a rotor-gene apparatus (Qiagen, Hilden, Germany). Each reaction contained 5 µL of QuantiNova SYBR Green PCR MasterMix and 1 µL of 1:8 dilution of cDNA as the template (total volume: 10 µL). The primers used for qPCR, their final concentration on the assay, and the annealing/extension temperature for each primer pair are listed in Supplementary Table S3. Internal standard genes for relative quantification (*sarA* and *cox5*) were selected according to a previous standardization for *Aspergillus niger* [37]. Relative expression of *sih1*, *sih2*, *sih4*, and AsHog1 genes was calculated as described by Pfaffl [38] using the REST software (2009, Qiagen, Hilden, Germany). 

### 2.8. Electron Microscopy and TEM Image Analysis

Fungal tissues were embedded in agarose gel to facilitate fixation procedures, as described elsewhere [39]. Briefly, the samples were fixed for 24 h in 2.5% glutaraldehyde in 0.1 M phosphate buffer (pH = 7.4). NaCl was added to the fixative to maintain osmolarity. Samples were washed three times with 0.1 M phosphate buffer and decreasing NaCl concentrations to avoid the collapse of cell structure due to osmotic shock. After washing, samples were post-fixed in 1% aqueous solution of osmium tetroxide for 1 h at 4 °C and embedded in Agar 100 resin (Structure Probe, Inc Supplies, West Chester, PA, USA). Ultrathin sections were contrasted with 1% aqueous solution of uranyl acetate and saturated solution of lead citrate in 0.1 M NaOH prior to observation with a CM 100 transmission electron microscope (Philips, Eindhoven, The Netherlands). To assess the ultrastructural differences in the cell wall composition between cells grown in different conditions of salinity, 50 cell wall measurements were performed per group. All measurements were performed on perpendicular cross-sections of comparable-sized hyphae by using Digital Micrograph software v2.11 (Gatan Inc. Pleasanton, CA, USA) and statistically evaluated by Student’s *t*-test.

### 2.9. Quantification of Compatible Solutes

For the determination of glycerol and trehalose in salt-adapted cultures of *A. sydowii*, the fungus was cultured for 7 days at 150 rpm and 28 °C in 250 mL flasks containing 100 mL of Vogel’s medium (without NaCl (No NaCl) and with 0.5 M NaCl or 2.0 M NaCl) supplemented with 2% glucose as the carbon source. The mycelium was collected by filtration through a 40 μm cell strainer and dried in an oven at 60 °C. Compatible solute extraction for HPLC analysis (Waters 600, Milford, MA, USA) was performed as previously described with minor modifications [40]. Briefly, 100 mg of dried mycelium was suspended in 3680 μL of chloroform:methanol:water solution (10:5:3.4) and stirred vigorously for 30 min. After that, 433 μL of chloroform and 433 μL of demineralized water were added, and the suspension was incubated for 30 min with stirring. The samples were centrifuged at 5500× *g* for 10 min for phase separation. The upper methanol:water phase was transferred to a 15 mL falcon tube and stored at −20 °C. The HPLC analysis was performed in an isocratic system with an AMINEX-HPX87H column (Bio-Rad, Munich, Germany, 300 mm × 7.8 mm) at 50 °C. Fifty microliters of each sample were injected, and 5mM sulfuric acid was used as the mobile phase at a flow rate of 0.8 mL/min. Glycerol and trehalose standards with an initial concentration of 5 mg/mL were used to construct calibration curves. Chromatogram analysis was performed using ChromQuest software version 2.51 (Thermo de Quest, Thermo Fisher Scientific, Waltham, MA, USA).

### 2.10. Data Availability

Sequencing short read data and processed data were deposited to SRA database under accession number GSE139804, BioProject PRJNA587059. All scripts used for the analysis of high-throughput sequencing data are available from the authors upon request.

## 3. Results and Discussion

### 3.1. Identification of a Terrestrial Isolate of Aspergillus sydowii

The strain BMH-0004 was isolated from a sugarcane bagasse fermentation with 2.0 M NaCl and a posterior screening on a solid medium with carboxymethylcellulose at 0.5 M NaCl [20]. This strain was initially classified as *Aspergillus caesiellus* by a polyphasic identification based on: (i) molecular markers (ITS, SSU, and LSU); (ii) micromorphological traits of the colonies, such as shape, size, and grouping of conidiophores and hyphae; and (iii) macromorphological aspects, such as exudates and pigmentation. Although none of the listed molecular markers rendered a resolved phylogram, the authors identified the strain as *A. caesiellus*, primarily based on micromorphology.

**Figure 1:**
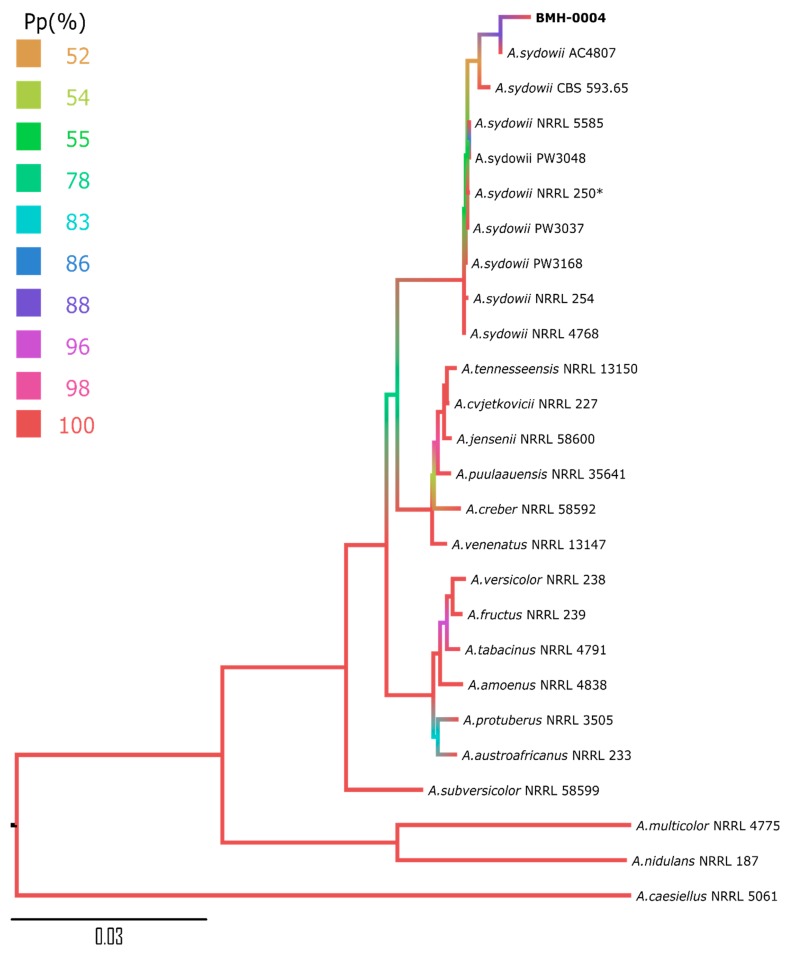
Bayesian phylogram obtained based on the concatenated alignment using an internal transcribed spacer (ITS), *benA*, *cam*, and *rpb2* from strains belonging to *Aspergillus* section *Versicolores* [44]. The color of the branch corresponds to the posterior probabilities (Pp) percentage. The accession number of the genes used for the phylogram is given in Supplementary Table S1. * *Aspergillus sydowii* reference strain [21].

Since the sequences of different parts of rRNA genes did not provide a clear classification of this fungus, additional DNA barcoding markers were used. Although ITS, SSU, and LSU are commonly utilized as primary fungal markers, within the *Aspergillus* genus, secondary markers are necessary to ascertain species level with higher confidence [21,41]. Therefore, *benA*, *cam*, and *rpb2* phylogenetic markers were also amplified to aid with identification. The reconstructed phylogeny with these markers showed that strain BMH-0004 is closely related to *A. sydowii*, *Aspergillus* section *Versicolores* [42], and is relatively distant from *A. caesiellus* (Figure 1 and Supplementary Figure S1), which is a member of *Aspergillus* section *Restricti* [43]. In comparison to the *A. sydowii* type strain CBS 593.65, all obtained nucleotide sequences of the strain BMH-0004 were highly similar: 100% identity in *benA* and 99 % in *cam* and *rpb2*.

**Figure 2:**
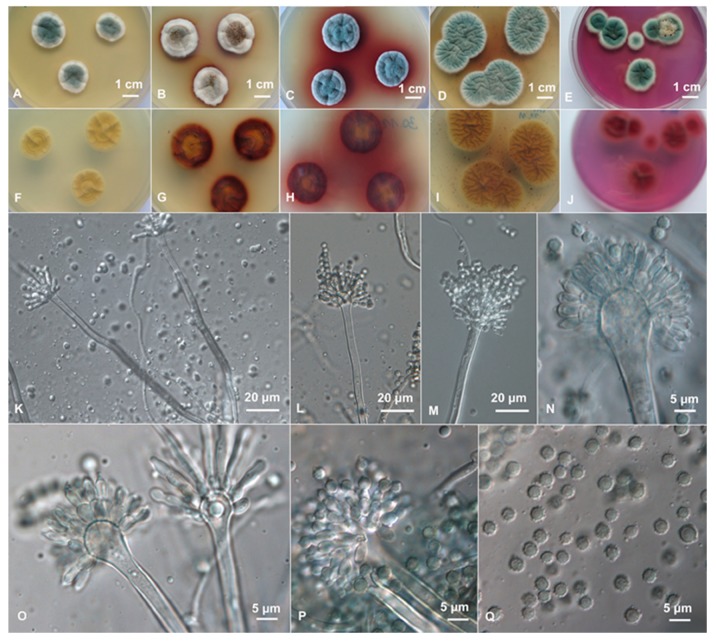
Macro and micromorphology of *A. sydowii* strain BMH-0004. (**A**) malt extract agar (MEA) 25 °C, (**B**) Czapek yeast extract agar (CYA) 25 °C, (**C**) CYA 30 °C, (**D**) CYA with saline (CYAS) 25 °C, and (**E**) creatine-sucrose agar (CREA). (**F**–**J**) Reverses in the same order as (**A**–**E**,**K**–**Q**) Conidiophores and conidia on MEA.

The morphology of this strain confirmed the characteristics of *A. sydowii* (Bain. and Sart.) Thom and Church [10]. It is mostly bluish-green and produces brown soluble exudates and brown soluble pigments when growing on CYA (Figure 2). Growth on other culture media used for *Aspergillus* identification is in accordance with *A. sydowii*. Additionally, microscopic parameters, like biseriate aspergilli, spathulate to subclavate vesicles measuring 10–20 μm, and cylindrical short neck phialides, 5–7 × 3 μm, as well as conspicuously spinose conidia of diameter 3–4 μm, are corresponding. The strain BMH-0004 could grow on solid and in liquid media without NaCl, but its optimal growth was detected when salinity ranged between 0.5 M and 1.0 M NaCl. The maximum tolerated salinity was 2.0 M NaCl when grown on glucose (unpublished data) and complex substrates (carboxymethylcellulose or wheat straw) [20].

### 3.2. Thriving and Enduring Require a Different Set of Adaptations

**Figure 3:**
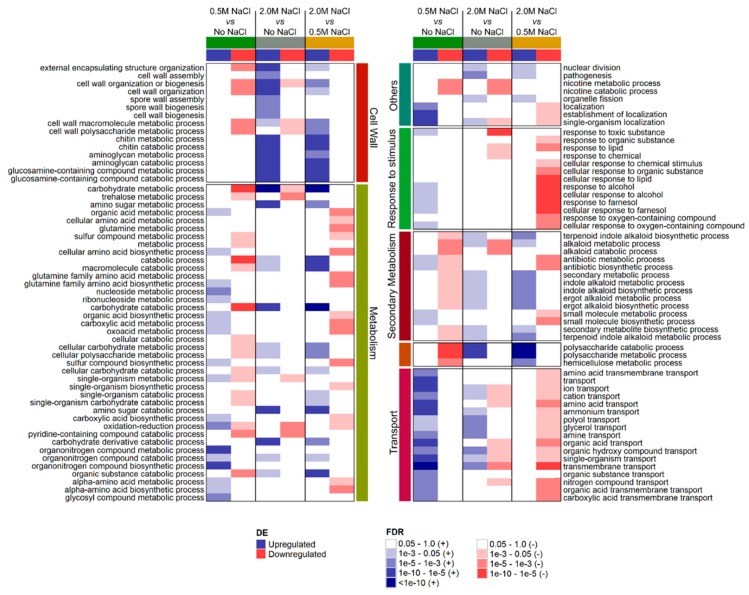
GO enrichment in the sets of differentially expressed (DE) transcripts for each evaluated comparison. False discovery rate (FDR) for the enrichment of each GO term was obtained by over-representation analysis.

Most studies on halophile physiology have been conducted in settings where the cells are exposed to hyperosmotic stress [14]. Under such circumstances, the physiological responses can be largely influenced by cellular stress-protective mechanisms. Actually, the clustering of expression level profiles of the transcriptomic data (Supplementary Figure S2) showed that the *A. sydowii* transcriptomes of the fungus growing without NaCl or at 2.0 M NaCl (nonoptimal conditions) were more similar to each other than to the transcriptome at the optimum salinity (0.5 M NaCl). This suggests that the expression profile under nonoptimal conditions is driven by stress-related responses, which include a global upregulation of central catabolic and secondary metabolism transcripts and substrate-degrading enzymes (Figure 3). Salinity-related responses differed between both salinity conditions. Under optimal salinity (i.e., 0.5 M NaCl), the fungus increased the expression of cation transport systems, presumably to maintain cellular ionic balance, but no other salinity related GO was enriched in this condition. Additionally, the energy strategy of the cells shifted towards an increase of the transcripts coding for solute transporters and a reduction of the transcripts for substrate degradation enzymes (Figure 3). In the case of hyperosmotic stress, cation transport systems are not pivotal (and indeed, the number of differentially expressed genes decreased when compared to 0.5 M NaCl), as the main challenge was osmotic, and therefore, the fungus overexpressed several transcripts associated to the remodeling of the fungal cell wall. The seemingly different strategies to cope with salinity lead to the question of whether some of the mechanisms described for halophiles are a consequence of saline stress and may not occur under optimal saline conditions. The main salt-response mechanisms described in other microorganisms are present in *A. sydowii*, although some differences were observed as well, and are discussed below.

### 3.3. Membrane and Cell Wall Are the Main Defenses under Saline Stress

In *A. sydowii*, there were no major changes in the transcription of genes involved in modifying the plasma membrane composition, a mechanism described as a response to salt in halotolerant fungi [45,46]. While the number of differentially expressed genes in the corresponding GO category was not significant, overexpression of two genes was observed: a Δ12-fatty acid desaturase at 2.0 M NaCl (logFC _2.0 M NaCl vs No NaCl_ = 2.12) and a methyl-sterol monooxygenase at 0.5 M NaCl (logFC _0.5 M NaCl vs No NaCl_ = 2.29). The overexpression of the former would imply an increase in unsaturated lipids, while the latter is involved in the ergosterol synthesis pathway. The action of these enzymes possibly increases membrane fluidity, which was observed previously in the extremely halotolerant black yeast *Hortaea werneckii* and the halotolerant black yeast *Aureobasidium pullulans* [45].

Transcriptional changes of genes associated with construction, deconstruction, and remodeling of the fungal cell wall suggest that, in *A. sydowii*, this structure is extensively modified in salinity conditions (Figure 3). The current model of the cell wall in *Aspergillus* describes that chitin, a linear polymer of β-1,4-*N*-acetyl-glucosamine, is located nearest to the cell membrane and cross-linked to α(1-3)-glucans forming a rigid hydrophobic core surrounded by cross-linked β(1-3)-glucans [47,48,49]. Covering this stiff region, a second layer of more hydrated β-glucans can be found. Finally, the outermost region of the cell wall is composed of mannan, arabinan, α(1-3)-glucans, and proteins [49].

**Figure 4:**
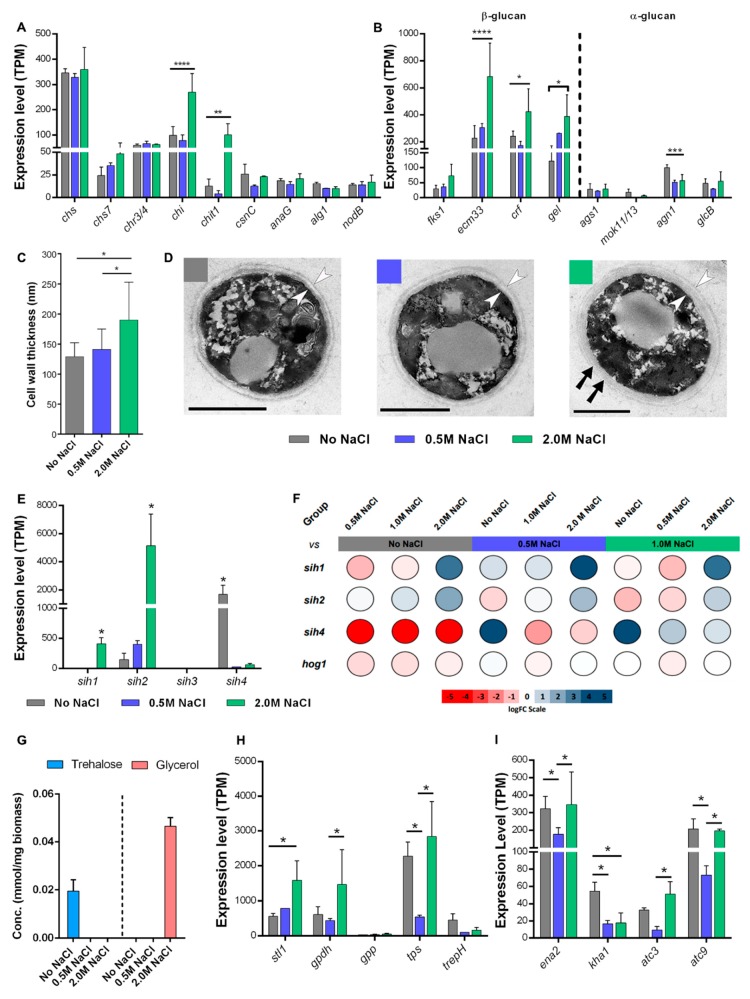
Mechanisms of tolerance to salinity and hyperosmotic stress identified in *A. sydowii*. (**A**) Cumulative expression level of transcripts associated to chitin/chitosan synthesis and degradation. (**B**) Cumulative expression level of transcripts associated to synthesis and degradation of glucans of the cell wall. (**C**) Cell wall thickness of comparable-sized hyphae. Results depicted as the averages and standard deviation of 50 measurements in each condition. Statistically significant differences (* *p* < 0.05) were identified by Student’s *t*-test. (**D**) Ultrastructural analysis of cross-sections of *A. sydowii* hyphae, showing lamellar structure (black arrows) and considerably thicker cell walls (arrowheads) in the samples grown in 2.0 M NaCl. Scale bars = 1 µm. (**E**) Expression levels (TPM) of hydrophobins identified in *A. sydowii* transcriptome. TPM: transcripts per million. (**F**) Relative quantification of *sih1*, *sih2*, *sih4*, and *hog1* gene expression levels by qPCR in cells grown without NaCl (No NaCl) or with 0.5 M, 1.0 M, and 2.0 M NaCl. Biological samples (*n* = 3) were assayed with two technical replicates. Color scale represents the logarithm of the expression fold change. (**G**) Glycerol and trehalose accumulation in the mycelium of *A. sydowii* when growing at different salinities. (**H**) Cumulative expression level of selected transcripts associated to the synthesis and degradation of compatible solutes. See main text for details. (**I**) Cumulative expression level of selected transcripts associated to metal cation transport. See main text for details. Statistically significant differences in (**A**,**B**,**E**,**H** and **I**) (**** *p* < 0.0001, *** *p* < 0.001, ** *p* < 0.01, and * *p* < 0.05) were identified by multiple Tukey’s tests.

The amount of chitin in cell walls is a function of its synthesis and degradation. Chitin synthases (CHS) are membrane proteins that polymerize intracellular UDP-N-acetyl-glucosamine and extrude the chitin polymer to the cell wall space. In *A. fumigatus*, eight CHS (*chsA*–*G*) genes have been identified, but their specific biological roles are still unclear, as deletion of many of these genes had no observable effect on growth or chitin content in the cell wall [50]. In *A.sydowii*, at least 11 different transcripts encoding eight chitin synthases genes (*chs*) were identified, but only one of these was strongly downregulated (logFC _2.0 M NaCl vs No NaCl_ = −3.18) when growing in the presence of salt. Nonetheless, the cumulative transcription level of all *chs* genes did not change significantly, suggesting that chitin synthesis is not affected (Figure 4A). Additionally, transcripts involved in the remodeling or modification of chitin/chitosan fibers, such as the *alg1* and *nodB* genes participating in mannosylation and deacetylation, were not differentially expressed. Instead, the endochitinase (*chi*) and chitotriosidase (*chit*) transcripts levels were upregulated when the fungus grew in 2.0 M NaCl, which may result in an overall decrease of the chitin and chitosan content in the cell wall.

As shown in *A. fumigatus*, *chsE* and *chsG* deletion mutants displayed substantial changes in growth, hyphal morphology, conidiation, and chitin content, with a decrease in chitin being counterbalanced by an increase in α(1,3)-glucan [51]. Interestingly, the upregulation of transcripts involved in the synthesis and modification of β-glucans in *A. sydowii* suggested that, in response to salinity, the increase in β-glucans was more pronounced than that of α-glucans (Figure 4B).

The cell wall of *A. fumigatus* is mainly composed of β(1,3)-glucans and α(1,3)(1,4)-glucans. The β (1,3)-glucan is synthesized as a linear polymer by the FKS1 synthase complex, while the extruded chains can be transferred to extend existent β(1,3)-glucan or to GPI-anchored proteins that link cell wall polysaccharides to the cell membrane. Four families of GPI-anchored proteins (ECM33, GEL1-7, CRH1-5, and DFG1-7) were found to be common to all fungi [52,53]. Genes from three of these families were upregulated at the highest salinity in the *A. sydowii* transcriptome, indicating the importance of cross-linkage modifications for maintaining cell wall integrity during osmotic stress, as discussed below.

ECM33 proteins are involved in the correct assembly of the cell wall β(1,3)-glucan and the mannoprotein layer [54]. Several mutants of the *ecm33* gene display increased sensitivity to a variety of stress conditions, such as oxidative agents, fungicides, cell wall perturbing agents, and osmotic and ionic imbalances [55,56]. ECM33 are assumed to participate in the crosslinking of β(1,3)-glucans to other cell wall components, although their precise biochemical function and their role in cell wall dynamics are not fully understood. The cumulative expression level of *ecm33* genes in *A. sydowii* was almost two times greater at 2.0 M NaCl than at the other two tested conditions, which indicates they favor the fungus’ stress tolerance.

The CRH glycosidases are responsible for transglycosylation, forming chitin-glucan linkages [57]. In Δ*crh* mutants of *Saccharomyces cerevisiae*, the chitin fraction is completely devoid of linkages to β-glucans [57]. Deletion of *crh* genes in *Candida albicans* increases the elasticity of the cell wall, reducing survival during osmotic shock, while overexpression of one *crh* gene has an osmoprotective effect [58]. Such protection might also happen in *A. sydowii*, where a CRH homologue gene (*crf*) was overexpressed at 2.0 M NaCl (logFC _2.0 M NaCl vs No NaCl_ = 1.23) (Figure 4B).

Finally, the GEL family of glucanosyltransferases is the most studied among cell wall-associated proteins, and its members are present in a relatively high amounts in fungal cell walls. Their function is to cleave the newly synthesized linear β(1,3)-glucan and transfer it to another β(1,3)-glucan molecule, resulting in elongation or shortening of the glucan fibers. GEL proteins also regulate the cross-linking of proteins into the cell wall, therefore allowing the correct assembly of cell wall structures [59]. In *A. fumigatus*, seven *gel* genes have been identified, of which only three (*gel1*, *gel2*, and *gel4*) are expressed during mycelial growth [50,60]. In this fungus, *gel4* is an essential gene [60], whereas Δ*gel2* mutants have reduced growth, and *gel1* deletion has no evident effect on morphology [61]. In *Neurospora crassa*, individual deletions of *gel* genes do not influence stress sensitivity [59], indicating that there is higher redundancy in their function than in *A. fumigatus*. Noticeably, if the function of GEL proteins is abolished—with a triple mutation in *N. crassa* [59] or a single mutation of the GEL homolog gene *gas1* in *S. cerevisiae* [62]—the chitin content in the cell wall is increased. This could be a compensatory mechanism to restore the rigidity of the cell wall, indicating a balancing effect between β(1,3)-glucan crosslinking and chitin production. In *A. sydowii*, the same balancing effect can be observed. At 2.0 M NaCl, chitin content should be lower due to increased chitin degradation, which is compensated by β(1,3)-glucan cross-linking as a result of higher GEL expression.

Our results indicate that, under hyperosmotic stress, the chitin content is reduced, while the content of β-glucans either increases or they are rearranged in the cell wall mesh. As evidenced in other organisms, these modifications (including the increased cross-linkage) generate a reduction in cell wall elasticity that allows resistance to external insults. Ultrastructural analysis of *A. sydowii* supported this notion, showing that the cells grown in 2.0 M NaCl exhibit significantly thicker cell walls compared to the other two investigated conditions (Figure 4C). Furthermore, the cell walls of the hyphae under hyperosmotic stress showed a distinctive lamellar structure when compared to the more uniform cell wall structure in the cells growing in 0.5 M NaCl or without salt (Figure 4D).

### 3.4. Coating for the Occasion

One of the novel halophilic strategies described in fungi is the production of hydrophobins (HFBs), which have been observed in both basidiomycete and ascomycete model fungi when growing in high salinity. HFBs are small extracellular proteins produced exclusively by filamentous fungi, which means that even yeasts do not contain genes for these proteins. HFBs self-assemble at hydrophobic-hydrophilic interfaces and have been associated with the morphogenesis of aerial hyphae and the adhesion of hyphae to hydrophobic surfaces [63]. The abundance of HFB-encoding genes in some fungal genera and the few studies on their physiological role suggests that HFB could fulfill additional functions besides those currently known.

Individual species of filamentous fungi usually contain between two and seven HFB genes [64]. In the genome of the obligate halophilic *Wallemia ichthyophaga*, there are 26 HFB genes, more than twice the number found in related species of the genus. At the low-salinity growth limit of this species (10% NaCl), eight HFB genes are overexpressed, while four are overexpressed at high salt concentrations (30% NaCl) [65]. Another interesting feature of HFB from halophilic fungi is that they have a higher percentage of acidic residues than their homologs in nonhalophilic fungi, which is a characteristic found also in other proteins from extremophilic archaea [65]. This suggests that HFB could have a relevant role in the mycelial growth under halophilic conditions, which is why the term “halophilic hydrophobins” was coined [66], but this is still to be proven.

Particularly in the genus *Aspergillus*, the estimated number of HFB per species is between six and 10, with an average of nine [67]. In the *A. sydowii* transcriptome, we identified four HFBs (*sih1-4*), fewer than in some other members of the genus. Of these, only three (*sih1*, *sih2*, and *sih3*) were completely represented in the sequenced genome of *A. sydowii*. In the genomic locus corresponding to the *sih4* transcript, there is a truncated version of the gene that codes for a protein product with only four cysteine residues. As this transcript was highly expressed when the fungus was grown without NaCl, we cloned the *sih4* coding sequence from cDNA and confirmed that the transcript corresponds to a complete HFB gene (data not shown). This gene might have been lost (or repurposed) during the migration of *A. sydowii* from the terrestrial to the marine habitat, which has been previously proposed as a mechanism of evolution of HFB genes in the ascomycete family of Hypocreales [68]. It also agrees with the findings that gene loss is more abundant in highly polymorphic gene families, because these do not fulfill essential functions for cell viability [69].

The main conserved characteristic of hydrophobins is the presence of eight cysteines with a relatively conserved spacing between them. This pattern was also present in the HFB sequences identified in the transcriptome (Table 1). According to the proposed classification based on cysteine spacing, only SIH2 could be classified in Class I. However, the Kyte-Doolittle hydropathy profile indicates that all sequences belonged to Class I (data not shown). *A. sydowii* hydrophobins do not have a high proportion of acidic residues, as observed for their orthologues in the halophilic *W. ichthyophaga*, and their pI values do not distinguish them from their counterparts in nonhalophilic fungi (Table 1).

Relative expression values (TPM) indicated that only *sih1*, *sih2*, and *sih4* were expressed under the culture conditions in which this fungus grew, whereas the levels of *sih3* were close to zero (Figure 4E). Relative quantification by qPCR confirms that *sih4* was expressed in the absence of NaCl, and its expression was almost abolished under salinity conditions (Figure 4F). On the other hand, *sih1* and *sih2* were expressed in the presence of 2.0 M NaCl, but their transcription levels in 0.5 M NaCl were very low. The differential expression of the HFB-encoding genes of *A. sydowii* suggests that these proteins might function as effectors under stress or nonoptimal growth conditions. Such a hypothesis is not unprecedented, as evidence of this particular function of HFBs has come to light in recent years [70], but their role during stress remains poorly understood. HFBs could facilitate a more dense packing of the mycelium due to increased hydrophobicity [71], thus protecting cells inside the pellet from the external insults. Additionally, more hydrophobic hyphae or spores would spread more efficiently in aqueous media, which could constitute an ecological strategy to increase the chances of escape from stressful conditions. These two scenarios do not account for the fact that the fungus expressed different HFBs under different stress conditions, leaving questions about the function of HFBs in hyphae development and stress response for future studies.

### 3.5. Compatible Solutes

Among the most crucial adaptations to salinity in fungi is an increased concentration of intracellular compatible solutes. For example, in *H. werneckii* and *W. ichthyophaga*, glycerol is the most abundant osmolyte, but also, erythritol, arabitol, and mannitol can be found in lesser amounts [40,72]. Similarly, in *A. niger*, glycerol and erythritol accumulate in young mycelium, whereas mannitol and erythritol are the signature compatible solutes in old mycelium [73,74]. In conidia of *A. niger* and *A. nidulans*, trehalose and mannitol have been found [75,76].

As mentioned earlier, in *A. sydowii* BMH-0004 strain, some transcripts associated with the metabolism of osmolytes were differentially regulated in response to osmotic stress. Noteworthy was the expression of genes involved in trehalose synthesis, where α-trehalose phosphate synthase (TpsA) was expressed under both hypo- and hyperosmotic stress (Figure 4H) and trehalose phosphorylase (TrePH) only under hypo-osmotic stress (i.e., without NaCl). Quantification of trehalose in *A. sydowii* shows that this metabolite was produced exclusively when the fungus was grown without NaCl (Figure 4G), indicating that TpsA expression alone cannot ensure trehalose accumulation. While, in *A. nidulans*, trehalose accumulates in response to oxidative and heat stress and during conidiation, the same is not the case for osmotic stress [77].

Conversely, there was an upregulation of genes related to the synthesis and transport of glycerol from the extracellular medium under hyperosmotic stress. This salinity caused an apparent upregulation (statistically significant only at cumulative expression, not at transcript level) of the glycerol-3-phosphate dehydrogenase gene (*gpd*), encoding the rate-limiting enzyme in the pathway of glycerol synthesis. At the same time, the transcription of the genes encoding the STL1 glycerol:H^+^ transporters, which facilitate the import of glycerol into the cells, also increased at 2.0 M NaCl. Glycerol was accumulated only at 2.0 M NaCl and not at the optimal salinity for growth (Figure 4G), indicating that, in *A. sydowii*, it is a signature osmolyte of stress. In many other halotolerant and halophilic fungi, accumulation of glycerol is thought to be mediated by an increase of the *gpd* transcript levels [78], although most studies have been performed exposing the organisms to short-term osmotic shocks. Meanwhile, in the long-term exposure to high salinity, the expression of *gpd* can be similar to nonsaline conditions, leaving the regulation of glycerol concentration to Stl1 transporters [79].

Osmotic stress activates a MAPK signaling pathway known by the name of its principal kinase: HOG (High Osmolarity of Glycerol). The activation of the HOG pathway induces the accumulation of glycerol, arrest of the cell cycle, and reorganization in the actin cytoskeleton, as well as changes in cell wall dynamics [80,81]. In this strain, we found two variants of the MAP kinase *hogA* that could regulate the pathways of adaptation to salinity conditions, but none was differentially expressed in response to salinity. Relative quantification of the main *hogA* gene variant by qPCR confirmed that this kinase is not changing its expression even under hyperosmotic shock (Figure 4F).

### 3.6. Membrane Transporter Expression Is Not Pivotal for Eluding Ion Toxicity

Although not universally, some fungal halophilic strains have a higher number of transport systems—when compared with mesophilic relatives – to regulate the homeostasis of alkali cation levels, as well as the fluxes across the plasma membrane, to eliminate toxic ions such as sodium (Na^+^) [65,82,83]. For this reason, we compared the total number of transporter genes in the genomes of five Aspergilli: the halophiles *A. sydowii* and *A. versicolor*, and the halotolerant relatives *A. nidulans, A. niger*, and *A. fumigatus* (Table 2). The halophiles *W. ichthyophaga* and *H. werneckii* were included as a reference. In the analyzed genomes, the total number of genes encoding AA uptake transporters (Pfam 01490 and 00324); MFS members (Pfam 07690 and 00083); and cation/transporter ATPase exchangers (Pfam 00689, 00690, and 01061) showed striking differences (Table 2). The genomes of *A. sydowii* and *A. versicolor* (halophiles) revealed a higher number of genes encoding APC members, which could have a role in ensuring a rapid uptake of certain amino acids under hypersaline conditions. Osmoregulation at increased salinity in prokaryotes often involves accumulation of amino acids, such as proline and betaines, which could be also the case in halophile fungi growing in rich substrates. The contribution of the APC transporters to halotolerance remains underexplored in filamentous fungi.

Gene expression analysis showed an enrichment of transport-related genes when *A. sydowii* was grown at 0.5 M NaCl but not at 2.0 M NaCl (Figure 3). In total, 137 and 94 transcripts predicted to transport cations according to TCDB/TrSSP analysis were upregulated at 0.5 M NaCl and 2.0 M NaCl against the condition without NaCl, respectively. However, most differentially expressed transcripts are not involved in maintaining the metal cation balance of the cell but are participating in the internalization of sugars, aminoacids, and other solutes resulting from the degradation of the wheat straw. These results, although counterintuitive, are similar to the findings in the halophilic basidiomycete *W. ichthyophaga*, where only three transcripts related to alkali metal cation transporters were overexpressed at different salinities [65]. One possibility is that nonmetal cation transporters, seemingly not related to salinity, are involved in osmotic balance when the fungus is growing optimally. Additionally, the expression level of cation transporting genes was low, which makes it difficult to assure the biological relevance of the estimated differential expression. For example, transcripts from a Na^+^/H^+^ antiporter *nah2* gene had a high logFC _0.5 M NaCl vs No NaCl_ (3.21), but their expression level mean across all conditions was 1.36 tpm.

The *ena2* gene (ATN2), a sodium P-type ATPase commonly associated with salinity adaptations in the yeast *Saccharomyces cerevisiae* and other fungi, was downregulated at 0.5 M NaCl when compared to the conditions without salt or 2.0 M NaCl. This gene was found differentially expressed in *W. ichthyophaga* and *H. werneckii* under high salinity conditions [65]. Our results indicate that, in *A. sydowii*, the expression of *ena2* is regulated by stress signals rather than high concentrations of salt. ENA P-type ATPase family in fungi are functionally different from animal Na^+^, K^+^- ATPases, as they have lower selectivity to cations (i.e., they pump out both alkali cations) [84]. In conditions with low Na^+^ concentrations, the ENA P-type ATPase pumps out K^+^ to maintain the Na^+^/K^+^ ratio, whereas the opposite occurs in hypersalinity conditions. Supporting this notion, the K^+^/H^+^ antiporter *kha1* gene, which presumably extrudes K^+^ from the cell or into intracellular vesicles [85,86], was overexpressed in the condition without salt. Finally, *atc3* and *atc9* genes are other putative P-type ATPases with the same expression profile as the *ena2* gene in *A. sydowii*, suggesting that the fungus actively transport cations under nonoptimal conditions to maintain the Na^+^/K^+^ ratio.

## 4. Conclusions

**Figure 5:**
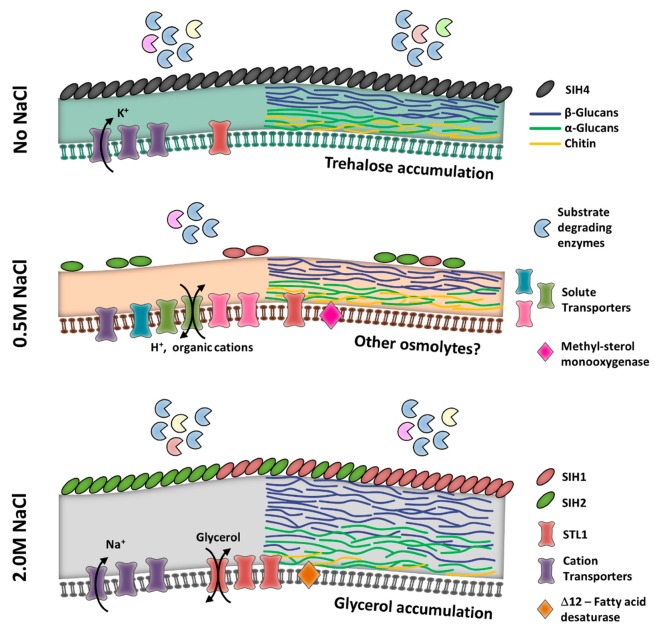
Proposed mechanisms of tolerance to salinity and hyperosmotic stress in *Aspergillus sydowii*. The location of SIH hydrophobins at the cell wall is putative. Cell wall composition was inferred from the expression of genes involved in the synthesis of polymers.

By comparing the transcriptomic profiles of *A. sydowii* in three different osmotic conditions, we learned that physiological responses to salinity vary greatly and are not a simple graded effect as the salt concentration of the medium is increased. In fact, in each condition assayed, the responses could be assumed as a distinct physiological state (Figure 5). When growing without NaCl, the cells of *A. sydowii* seem to be exposed to hypoosmotic stress, which elicits the production of trehalose and the overexpression of a hydrophobin gene (*sih4*) (Figure 4). Stress also triggers an increase in the transcription of genes for substrate degradation and carbohydrate catabolic processes, very similar to the effect in hyperosmotic conditions (i.e., 2.0 M NaCl). Oppositely, at optimal growth circumstances, the biosynthetic metabolic processes are favored, and the strategy of the cell is to increase transport systems for the efficient uptake of nutrients instead of the energy-demanding synthesis of extracellular enzymes. Osmotic balance in this condition is likely achieved by accumulating high energy-containing metabolites, such as mannitol, sorbitol, or arabitol. Only when the osmotic pressure increases up to nonoptimal conditions, the fungus starts to produce glycerol, reshape the cell walls and increase its thickness, possibly changes membrane composition, and produces a different set of hydrophobins (*sih1* and *sih2*).

While most of the mechanisms of adaptation to salinity described to date in eukaryotes are also occurring in *A. sydowii*, these are not taking place concurrently. The adaptations to ensure osmotic balance and avoid ion toxicity when the fungus grows under optimal salinity conditions are not completely understood, as they do not coincide with the halophile adaptations described in the literature. Further experiments should be conducted to identify and understand these adaptations and the effects of different stress signals on modulating the response. Our results indicate that the interpretation of physiological reactions in extremophiles or extremotolerant microorganisms should be addressed differently, as stress can reshape the physiological outcome. In this sense, the strain *A. sydowii* BMH-0004 was a useful model to observe these transitions.

## Figures and Tables

**Table 1:** Sequence properties of hydrophobins from *Aspergillus sydowii* BMH-0004.

Gene	Sequence Properties ^a^				Class	Cysteine Spacing				
	Length	pI	%Basic	%Acidic		C1-C2	C3-C4	C4-C5	C5-C6	C7-C8
*sih1*	129	3.98	5.4	10.9	Unclassified	6	35	19	5	19
*sih2*	137	4.05	3.7	7.3	Class I	7	39	18	5	17
*sih3*	110	4.02	6.4	12.7	Unclassified	5	32	6	5	13
*sih4*	117	3.99	7.7	17.1	Unclassified	6	30	25	8	4

^a^—of mature protein without signal peptide.

**Table 2:** Genomic distribution of transporter families within Aspergilli species *.

Transporter Family	Pfam Number	*A. sydowii*	*A. versicolor*	*A. nidulans*	*A. niger*	*A. fumigatus*	*W. ichthyophaga*	*H. werneckii*
Alkali metal cation/H+ antiporter	PF08619	1	1	1	1	1	0	**2**
Sodium: solute symporter	PF00474	5	**7**	4	4	3	2	6
MFS	PF07690 PF00083	447 171	**452** 164	271 106	335 96	220 83	52 17	342 **178**
Cation transporter/ATPase	PF00690 PF00689	**21** **16**	16 12	13 11	13 7	12 11	7 4	14 9
ABC Transporters	PF01061 PF00664 PF00005 PF06422	21 22 52 16	**22** 31 **75** **20**	18 24 49 14	21 **32** 62 16	15 23 52 12	3 10 20 2	14 **32** 8 10
AA uptake	PF13520 PF01490 PF00324	0 32 86	0 31 **87**	0 13 52	0 22 61	0 16 46	0 9 5	46 **33** 27
V-Type ATPase-I	PF01496	1	1	**2**	1	1	1	**2**
Na^+^/H^+^ exchanger family	PF00999	8	**10**	8	7	7	6	14
Na^+^/Ca^2+^ exchanger family	PF01699	8	7	6	9	7	3	**12**
Cation transporter family	PF02386	6	3	3	4	3	1	**8**

* The highest number of genes in each family is represented in bold.

## Supplementary Materials

The following are available online at https://www.mdpi.com/2073-4409/9/3/525/s1: **Table S1:** NCBI accession numbers of the genes used in the phylogenetic analyses. **Table S2:** Parameters for maximum likelihood trees using PhyML-SMS. **Table S3:** Primer and PCR conditions used in this study. **Figure S1:** Maximum likelihood phylograms based on their specific best substitution models. All the nodes with bootstrap support >50% are indicated at nodes with a circle. * *Aspergillus sydowii* reference strain [21]. **Figure S2:** Effect of filtering out the transcripts with low read counts on gene expression profiles and discrimination between experimental groups by unsupervised clustering (**a** and **b**) and the identification of DE transcripts (**c**).
