# Supplementary material for: Stress Reshapes the Physiological Response of Halophile Fungi to Salinity
Source: Cells. 2020 Feb 25;9(3):525. doi: 10.3390/cells9030525 (PMC7140475; doi:10.3390/cells9030525)

Supplementary Materials

Table SI. NCBI Accession number of the genes used in phylogenetic analyses**.**

Species	*tubß*	*cam*	*rpb2*	ITS
Aspergillus creber	JN853980	JN854043	JN853832	JQ301889
A. puulaauensis	JN853979	JN854034	JN853823	JQ301893
A. tennesseensis	JN853976	JN854017	JN853806	JQ301895
A.cvjetkovicii	EF652264	EF652352	EF652176	EF652440
A. jensenii	JN854007	JN854046	JN853835	JQ301892
A. venenatus	JN854003	JN854014	JN853803	JQ301896
A. versicolor	EF652266	EF652354	EF652178	EF652442
A. fructus	EF652273	EF652361	EF652185	EF652449
A. tabacinus	EF652302	EF652390	EF652214	EF652478
A. amoenus	JN853946	JN854035	JN853824	EF652480
A. austroafricanus	JN853963	JN854025	JN853814	JQ301891
A. protuberus	EF652284	EF652372	EF652196	EF652460
A. subversicolor	JN853970	JN854010	JN853799	JQ301894
A. multicolor	EF652301	EF652389	EF652213	EF652477
A. nidulans	EF652251	EF652339	EF652163	EF652427
A. sydowii NRRL 250 Reference	EF652274	EF652362	EF652186	EF652450
A. sydowii CBS59365	EF428373	EU443971	NA	AB267812
A. sydowii NRRL 5585	JN853936	JN854039	JN853828	NA
A. sydowii NRRL 4768	JN853935	EF652385	EF652209	EF652473
A. sydowii NRRL 254	EF652275	EF652363	EF652187	EF652451
A. sydowii PW3168	LC000553	LC000566	LC000579	AB987908
A. sydowii PW3048	LC000545	LC000558	LC000571	AB987900
A. sydowii AC4807	KJ413350	NA	KJ476438	KJ413376
A. sydowii PW3037	LC000544	LC000557	LC000570	AB987899

Table S2. Parameters for Maximum likelihood trees using PhyML-SMS

Loci	Substitution model	Log-likelihood	Discrete gamma model	Number of categories	Gamma shape parameter	G-Blocks?
tubß	HKY85	-1198.70048			0.331	Yes
cam	TN93	-2613.55927			0.35	No
rpb2	TN93	-3494.24192	Yes	4	0.215	No
Concatenated alignment based on 4 loci (*tubß*, *cam*, *rpb2* and ITS)	TN93	-10563.49438			0.685	-

Table S3. Primer and PCR conditions used in this study.

Gene/Marker	Primer name	Sequence	Length (nt)	Tm(°C)	Th(°C)	Conc. (nM)	Purpose
ITS	ITS1	TCCGTAGGTGAACCTGCGG	19	63	55	200	Phylogeny marker
	ITS4	TCCTCCGCTTATTGATATGC	20	54		200	
rpb2	RPB2-5F	GAYGAYMGWGATCAYTTYGG	20	47-61	55	200	Phylogeny marker
	RPB2-7CR	CCCATRGCTTGYTTRCCCAT	20	55-65		200	
benA	Bt2a	GGTAACCAAATCGGTGCTGCTTTC	24	62	55	200	Phylogeny marker
	Bt2b	ACCCTCAGTGTAGTGACCCTTGGC	24	66		200	
cam	CMD5	CCGAGTACAAGGARGCCTTC	20	58-61	55	200	Phylogeny marker
	CMD6	CCGATRGAGGTCATRACGTGG	21	57-64		200	
sarA	Fw_qPCR_sar1	GTTGCGATCCTTTCTCCTACC	21	56	63	500	qPCR reference gene
	Rv_qPCR_sar1	ACAATTCCGCTAACTTCAGGG	21	56		500	
cox5	Fw_qPCR_cox5	TCTCTGTCGGCGTTTTCTAC	20	55	63	700	qPCR reference gene
	Rv_qPCR_cox5	AGAGCGTATTCGTTGGTAGC	20	56		700	
sih1	Fw_qPCR_sih1	CGTCGATGCTGATGTTCTTAAC	22	55	63	700	qPCR
	Rv_qPCR_sih1	ACGGGAATGCAAGGGATG	18	56		700	
sih2	Fw_qPCR_sih2	TGTCGGAAACACTGGTAACAG	21	56	65	500	qPCR
	Rv_qPCR_sih2	GTGACCTCGTTACAGCAAGAG	21	56		500	
sih4	Fw_qPCR_sih4	GAGGAAACCCAAGAGTACGAC	21	55	63	700	qPCR
	Rv_qPCR_sih4	TGTTGCCTCCGATAAGGTTG	20	56		700	
hog1	Fw_qPCR_hog1	TCGAGGTAGACATCTGGAGTG	21	56	63	700	qPCR
	Rv_qPCR_hog1	ACTGGTTAACGTGGTCTTTCC	21	56		700	
sih4	Fw_sih4	ATAGAATTCGAGCAGAAACTCATC-> TCTGAAGAGGATCTGATCGAAGGT-> CGTATGCCTTCCTCCGAGCAGGCC	72	79	58	200	*sih4* cds cloning – Forward primer includes a c-myc tag and a Factor Xa cleavage site
	Rv_sih4	ATTCTAGATTACTCCTCGGCCTCCTCGGTCT	31	72		200	

Abbreviations: **Tm** – Calculated Melting Temperature of primer, **Th** – Actual Annealing Temperature used in PCR, **Conc**. – Primer concentration in the assay.

Figure S1. Maximum likelihood phylograms based on their specific best substitution models. All the nodes with bootstrap support >50% are indicated at nodes with a circle. **Aspergillus sydowii* reference strain (Samson et al. 2014).

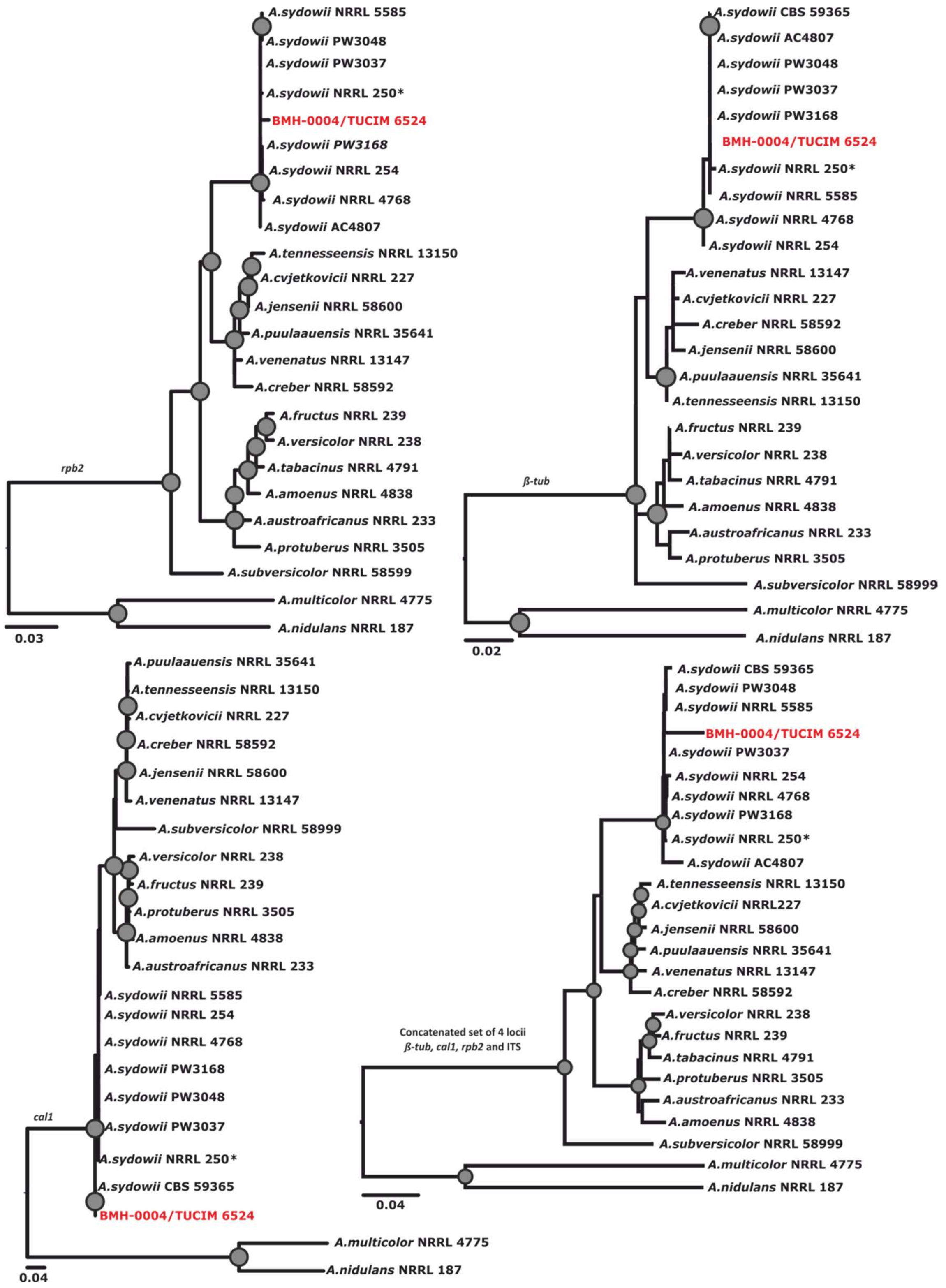

Figure S2. Effect of filtering out the transcripts with low read counts on gene expression profile and discrimination between experimental groups by unsupervised clustering (**a** and **b**) and on the identification of DE transcripts (**c**).

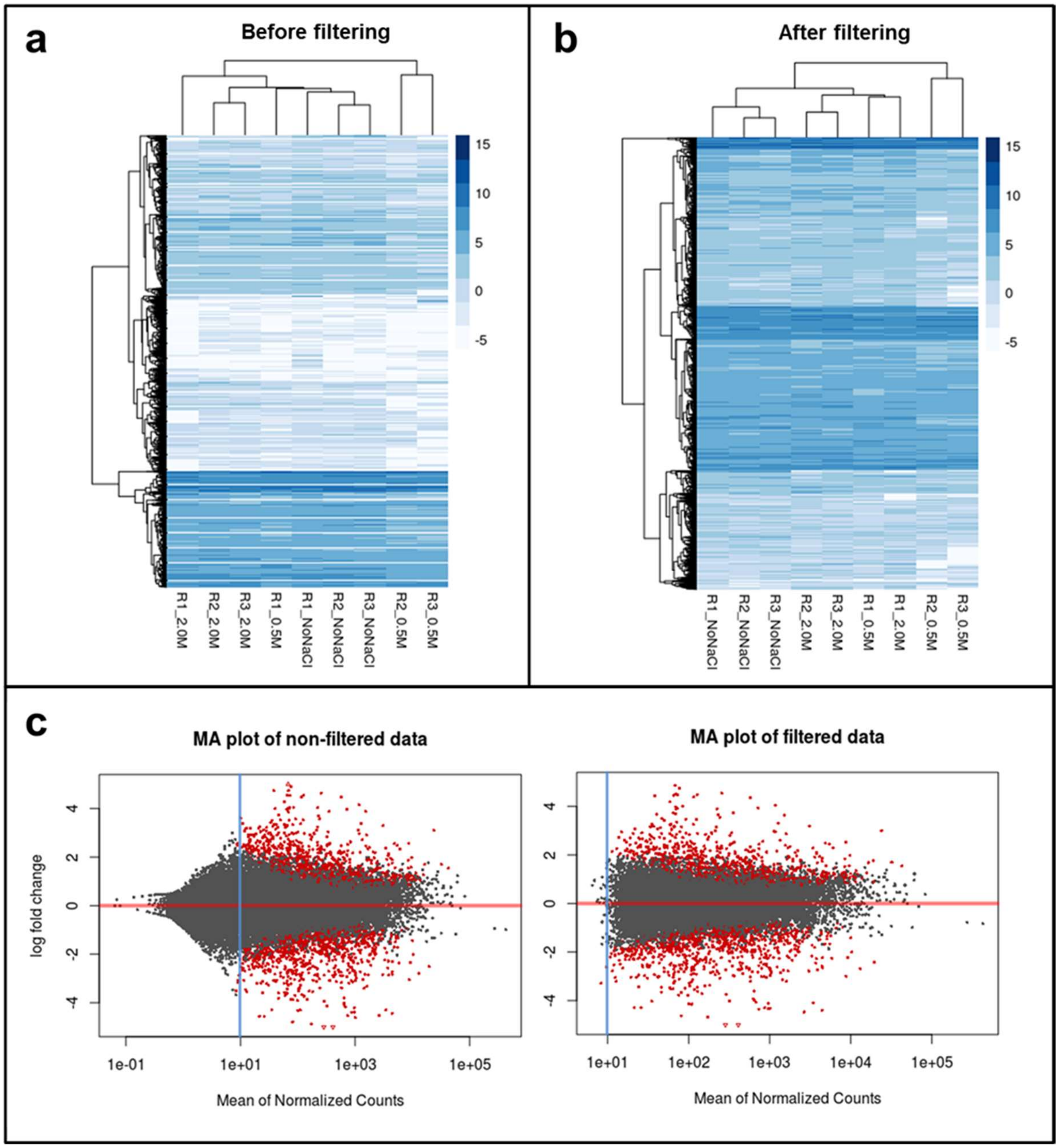

Supplement: Supplementary file 1 [file cells-09-00525-s001.pdf]
